# The Impact of Diet and Nutrition on Prostate Cancer – Food for Thought?

**DOI:** 10.1007/s11912-025-01641-x

**Published:** 2025-02-15

**Authors:** Rodrick Babakhanlou, Krisstina Gowin

**Affiliations:** 1 Division of Hematology & Oncology, The University of Arizona Cancer Center, Tucson, AZ USA; 2 Division of Supportive Care and Hematology HCT, The City of Hope, Orange County, CA USA

**Keywords:** Prostate cancer, Nutrition, Diet, Phytonutrients, Antioxidants, Proteins, Fats, Carbohydrates, Vitamins

## Abstract

**Purpose of Review:**

Prostate cancer is the second most common type of cancer in men.

Its incidence varies widely and is influenced by geographic location, race, ethnicity, lifestyle factors, and diet. The purpose of this review is to discuss the association between prostate cancer and diet and outline the impact of fats, carbohydrates, proteins, vitamins and phytonutrients on the pathogenesis of disease.

**Recent Findings:**

Although conclusive evidence is limited, current data is indicative that a diet low in particular fats, animal proteins, dairy products and high in vegetables and fruits can be beneficial in supporting the course of disease.

**Summary:**

Promoting a dietary pattern low in processed meat, dairy products, refined carbohydrates and saturated fats, but high in fruits and vegetables may have beneficial effects on prostate metabolism and inhibit various stages of carcinogenesis.

## Introduction

Prostate cancer is the second most common type of cancer in men and the fifth most common cause of cancer-related death worldwide [1]. Approximately 11.6% of all men will be diagnosed with prostate cancer during their lifetime [2]. Prostate cancer is most frequently diagnosed in men 65–74 years of age. The incidence varies widely and is influenced by geographic location, race, ethnicity, lifestyle factors and diet [2–4].

Although the etiology of prostate cancer is multifactorial, the influence of geographic and ethnic differences suggests that diet, environmental factors, lifestyle factors and metabolic problems may have a substantial impact on the development and progression of disease [3]. Epidemiological evidence indicates that high body mass index and obesity are not only associated with an increased risk of developing prostate cancer, but also with disease progression [2, 5]. There is a strong association between obesity and the Western diet. Indeed, the incidence of prostate cancer is six-fold higher in Western countries compared to non-Western countries with a low incidence in Asian countries [3, 6].

The role of lifestyle medicine is growing in prostate cancer and healthy dietary choices are known to positively impact both overall wellbeing and disease outcomes in this patient population.

This review will summarize and discuss associations between prostate cancer and diet and outline the impact of fats, carbohydrates, proteins, vitamins and phytonutrients on disease outcomes.

### Dietary Factors

#### Dietary Fats

Fats form part of every cell and are vital for energy, physiological and biological processes [7, 8]. In the body, lipids exist in various forms, each with a different structure and function, including fatty acids, triglycerides, cholesterol and phospholipids [3, 7].

Fatty acids are divided into saturated and unsaturated fatty acids. Unsaturated fatty acids are further divided into monounsaturated and polyunsaturated fatty acids (PUFA) [9].

Although the body can manufacture most of the fats needed, there are two groups of fatty acids that the body cannot manufacture [8]. These are termed essential fatty acids and include the omega-6 fatty acids and the omega-3 fatty acids [8]. Both the omega-6 fatty acids and the omega-3 fatty acids are PUFA.

While the optimal ratio between omega-6 and omega-3 fatty acids should be 3:1, the Western diet is abundant in omega-6 fatty acids with a ratio of up to 30:1 [10, 11]. Animal products, such as dairy and meat, are the primary source of monounsaturated, polyunsaturated and saturated fatty acids [12, 13].

Several clinical and preclinical studies have shown a correlation between obesity and diets rich in omega-6 fatty acids and chronic diseases, including cancer [13, 14]. A product of the omega-6 fatty acid group is arachidonic acid that is converted to prostaglandins and leukotrienes, which is associated with alterations of the tumor microenvironment, pro-inflammatory states, tumor growth, proliferation and invasion [10, 14].

Prostate cancer cells adapt their metabolism in response to changes in hormonal status, growth factors, nutrient supply and epigenetic factors [14]. It is believed that diets rich in omega-6 fatty acids and trans fatty acids result in increased androgen levels, growth factors, oxidative stress and free radical damage [13–16].

Animal studies in mouse models confirmed that omega-6 fatty acids promote the growth of prostate cancer cell lines and that a diet rich in omega-3 fatty acids resulted in significantly slower tumour growth rates and lower serum prostate-specific antigen (PSA) levels [11, 16–19].

A large, population-based cohort study investigating the impact of dietary fat on prostate cancer in 229 men emphasized the importance of the type of fatty acids on the risk of development and progression of prostate cancer, highlighting a significant association between the risk of prostate cancer and the intake of both saturated and trans fatty acids [6].

The mechanisms associated with the intake of both saturated- and trans fatty acids and prostate cancer include increased oxidative stress generated during fat metabolism, induction of inflammation within the prostate gland, increased serum testosterone levels and insulin-like growth factors (IGF) and free radical formation [3, 6, 20, 21]. Moreover, the increased consumption of those fatty acids affects hormone metabolism with a resultant increase in intraprostatic androgens, promoting tumor growth and metastases [3].

The consumption of long-chain omega-3 fatty acids, primarily by consuming oily fish, has been identified to have protective effects against prostate cancer [2, 3, 5, 22–25]. This is believed to be the result of a delayed conversion from androgen-sensitive to androgen-resistant cancer cells, suppression of tumorigenesis by lowering testosterone and androgen receptor levels and growth factors, but also through anti-inflammatory effects [2, 3, 22].

Inflammation plays an important role in the pathogenesis of prostate cancer and cyclo-oxygenase (COX)−1 and COX-2 levels are increased in prostate cancer patients [19]. Omega-3 fatty acids exert their anti-inflammatory effects by inhibiting pro-inflammatory mediators, such as leukotriene B4, interleukin (IL) 1β and IL-6 with modulation in COX-1 and COX-2 [10, 19, 25]. Moreover, diets rich in omega-3 fatty acids exert beneficial effects on the IGF- pathway, leading to prevention or even inhibition of malignant growth in prostate cancer cells [25].

In general, high intake in dietary fat is associated with increased risk of prostate cancer. A diet low in fat, particularly low in saturated fats may be beneficial [6, 25]. Food sources rich in omega-3 fatty acids may exert protective effects against prostate cancer [22–25].

### Flaxseed

Flaxseed oil contains alpha-linolenic acid (ALA) and linoleic acid (LA) and is a rich source of dietary lignans and phenolic compounds [26]. While ALA is an essential fatty acid with anti-inflammatory properties, lignans exert anti-mitotic, anti-angiogenic and antioxidant effects [26, 27]. In addition, plant lignans may inhibit tyrosine kinase and topoisomerase and interfere with cellular proliferation [26]. Furthermore, flaxseed oils increase natural killer cell activity, inhibit cell membrane synthesis and induce necrosis of cancer cells and apoptosis [27, 28].

In the prostate gland, flaxseed inhibits 5-alpha reductase, affecting cellular proliferation and differentiation [26].

Studies in animal models showed that 5% flaxseed supplementation inhibited the growth and progression of prostate cancer in mice [29]. After 30 weeks of treatment, prostate cancer cells in the flaxseed group were less aggressive compared to the control group [29]. Human studies are limited and controversial.

A presurgical trial of flaxseed supplementation (30g/d) for 30 days resulted in a significant increase in the urinary concentration of total enterolignans and enterolactone [30]. Enterolactone is the most abundant circulating lignan in humans and is produced after conversion of plant lignan glycosides by the intestinal microflora [31]. The results showed that total urinary enterolignans and enterolactone were inversely correlated with both Ki67 in tumor tissue and vascular endothelial growth factor (VEGF) [30].

In a Swedish study, high serum enterolactone concentrations were associated with decreased risk of prostate cancer, but the significance of serum enterolactone with localized or advanced prostate cancer was not persistent after adjustment of additional factors [32]. A short-term pilot study examined the relationship between plasma enterolactone concentration and prostate tumor cell apoptosis and proliferation in men consuming lignan-rich rye bread. After three week there was a significant increase in the apoptotic index in biopsies of prostate tumors in the rye bread group compared to the control. No significant changes were seen in the plasma PSA levels [33].

Although flaxseed oil has beneficial effects on prostate cancer cells, the ideal amount, dose and duration of treatment are not known, and more research is needed for further clarification.

#### Dietary Proteins

Proteins are macromolecular polypeptides composed of many peptide-bonded amino acids. The human body requires 20 amino acids for protein synthesis. There are nine amino acids the body cannot synthesize in sufficient quantities, which are termed essential amino acids. Therefore, humans are required to consume foods rich in those essential amino acids to support protein synthesis [34].

In general, humans consume proteins in form of animal proteins, plant-based proteins and dairy products [3, 34]. Foods rich in animal protein are meat, fish, poultry, eggs and dairy products, while plant-based diets rich in proteins include legumes, nuts and grains [35].

### Meat

There is evidence that high intake of certain types of protein, in particular animal protein, such as red meat and processed meat, and dairy protein, increases the risk of prostate cancer [3, 16, 19, 34, 36, 37].

In Western countries, the consumption of meat is one of the main animal-derived products in the diet [38, 39]. Animal meat is not only composed of protein, but also fat and cholesterol [39]. Certain ways of preparing and processing animal meat, such as cooking at high temperatures, grilling and barbequing can increase carcinogenesis [3, 37, 39]. Carcinogens include N-nitroso compounds and can be formed in meats preserved with nitrates or nitrites, such as in sausages or cured meat, or can be produced endogenously by the reaction of amines from red meat with nitrosating agents in the intestines [37]. Preparing meat at high temperatures may result in increased levels of heterocyclic amines. The prostate gland can metabolize those chemicals into activated carcinogens, resulting in DNA damage and prostate cancer carcinogenesis [37]. In rodents, heterocyclic amines were shown to induce inflammation in areas of the prostate where the tumour developed at a later stage and also contributed to the progression of disease [3, 37, 40]. Inflammation is associated with increased levels of IGF-1, which is a known potent mitogen for androgen-sensitive and androgen-independent prostate cancer, leading to dysregulation of the PI3K/Akt/mTOR pathway, which plays a key role in the pathogenesis of prostate cancer [16, 36, 40–43].

Anti-androgen therapy (ADT) is a mainstay of prostate cancer treatment by lowering endogenous levels of testosterone [41]. However, ADT also induces hyperglycemia and hyperinsulinemia, resulting in increased levels of IGF-1, further enhancing aberrant growth [41]. Since over 50% of men on long-term ADT will develop metabolic syndrome during their treatment, dietary therapy and low-protein intake may support the management of advanced prostate cancer through reductions of IGF-1 levels [41, 44].

### Fish

Fish consumption has been suggested to prevent both development and progression of prostate cancer [45]. Populations with high consumption of fish, such as those in Asia and Alaskan Eskimos have lower rates of prostate cancer compared to those, who adhere to Western diets [46].

The rationale for the preventative effects of fish on prostate cancer has been attributed to the content of long-chain omega-3 polyunsaturated fatty acids [45, 46]. The omega-3 fatty acids found in fish contain eicosapentaenoic acid (EPA) and docosahexaenoic acid (DHA). These fatty acids exhibit anti-inflammatory properties through their impact on prostaglandin synthesis [46]. There is a wide range of mechanisms by which omega-3 fatty acids exert anti-cancer effects. EPA and DHA have been reported to inhibit growth and progression of prostate cancer, induce apoptosis via the PI3K/AKT pathway and reduce oxidative stress and inflammation via inhibition of the COX and lipooxygenase (LOX) pathways [25, 46–50]. In the clinical setting, the use of omega-3 fatty acids in the context of prevention and treatment of prostate cancer remains controversial, since the results of numerous studies and systematic reviews have been inconsistent [25, 46–53].

In three meta-analyses, no significant association was demonstrated between total fish intake and a reduction in prostate cancer incidence. However, there was an association between fish intake and a reduction in prostate cancer-related mortality [50, 52, 54].

One prospective study demonstrated that consumption of fish more than three times per week was associated with a reduced risk of prostate cancer [25].

Those mixed results are caused by a significant heterogeneity among studies. There is marked variability in study methodologies, analysis and reporting of individual studies, the stage of disease and outcomes, the type of fish consumed and cooking and preparation methods [25, 46–53].

Oily fish, such as salmon, herring, mackerel, anchovies or sardines, is rich in omega-3 fatty acids and vitamin D, contributing to its anti-inflammatory and anti-cancer effects [46, 48]. The consumption of oily fish once per week or more was shown to be associated with a decreased risk of advanced prostate cancer [48].

The consumption of fried fish, smoked salmon or salted fish were shown to be associated with an increased risk of prostate cancer [46, 48].

Literature does not differentiate between farm-raised vs. organic wild caught fish. One important downside of farm-raised fish is its exposure to growth hormones, such as testosterone, and antibiotics, which may have a negative impact on disease outcome in patients with prostate cancer [55, 56]. Depending on the geographic location, wild caught fish harbors the risk of being contaminated with heavy metals, such as arsenic, cadmium, lead or mercury, and its consumption, even at a low rate, can result in a cumulative lifetime cancer risk [57].

In addition, the processing and packaging of fish can have long-term health implications, especially with the use of cans and plastic, and possible contamination with heavy metals and Bisphenol A (BPA) [58, 59].

The impact of those factors on disease outcome has not been explored in the literature and needs to be elucidated.

### Eggs

Eggs are frequently consumed worldwide and have a high nutritional value, being rich in vitamins, minerals, proteins, and mono- and polyunsaturated fats [60]. It is believed that the high content in cholesterol and choline may play a role in the development of sex-hormone-related cancers, such as prostate cancer [5, 60]. Cholesterol serves as a precursor for the biosynthesis of sex hormones, such as androgens, that promote cell proliferation. Choline has been reported to contribute to the proliferation and progression of prostate cancer through cell membrane synthesis [60–62]. The high protein content of eggs increases the production of IGF-1, which promotes tissue growth and tumor progression [41, 44, 60].

The results of numerous studies regarding the impact of egg consumption on prostate cancer have been inconsistent.

In a study of 971 men diagnosed with prostate cancer and treated with prostatectomy, the intake of one large egg per day was associated with the likelihood of high-grade disease. The authors reported a positive association with the intake of eggs and prostate cancer progression [63]. The Cancer of the Prostate Strategic Urologic Research Endeavor (CapSURE) showed that men, who consumed the most eggs after prostate cancer diagnosis (5 eggs/week), had a twofold-increased risk of prostate cancer recurrence compared to men, who consumed the least and were at increased risk of fatal prostate cancer [52].

On the other hand, a meta-analysis assessing ten studies was not able to demonstrate an association between egg consumption and the risk of total prostate cancer [60].

### Poultry

The consumption of skinless poultry is not associated with an increased risk of developing aggressive prostate cancer or progression of existing disease [5]. In contrast, increased consumption of poultry with skin (> 3 servings/week) is associated with both increased risk of advanced prostate cancer and prostate cancer progression [5, 52].

Men in the highest quartile of poultry intake had a four-fold increased risk of progression and were more likely to undergo radical prostatectomy as their primary treatment. A plausible explanation for this observation is the high concentration of heterocyclic mutagens in well-done poultry [52].

### Dairy-based Proteins

Dairy products, such as milk, cheese or yogurt, are another source of protein [39]. A significant consumption of dairy products, especially whole milk, has been proposed to increase the risk of prostate cancer [19, 38]. The exact components of dairy products leading to an increased risk of prostate cancer are unknown [39]. While some authors postulated that dairy protein is positively associated with increased IGF-1 levels, which may stimulate the initiation and progression of prostate cancer, others assumed the amount of dietary calcium and fat content to be relevant in the disease process [5, 19, 39].

Existing research regarding the association between consumption of dairy products and prostate cancer shows inconclusive results [36, 64–67]. A recently published overview of systematic reviews and meta-analyses with cohort studies and case–control meta-analyses showed inconsistent results, even though some of the analysed reports did demonstrate a positive relationship between a high intake of milk and dairy products and prostate cancer [64]. A recent meta-analysis explored the association between the amount of total dairy products, total milk, cheese, butter and yogurt to prostate cancer [65]. In the dose–response analysis, an increase in total dairy intake by 400g/d was positively associated with an increased risk of prostate cancer by 2%. Further more, a positive association between the risk of prostate cancer and total milk intake was observed. While the consumption of whole milk was inversely associated with the risk of prostate cancer, there was no association between the consumption of low-fat milk and prostate cancer. While no significant association was observed between cheese intake and prostate cancer, an increase in butter intake by 50g/d was associated with an increased risk of prostate cancer [65].

A meta-analysis of observational studies demonstrated an increased risk of prostate cancer with high intake of total dairy products, milk, cheese, dietary calcium, low-fat milk and skim milk combined. A positive association was observed for dietary calcium intake of > 1500mg/d and an increased of prostate cancer [66].

In a prospective study of 525 men with newly diagnosed prostate cancer, the consumption of high-fat milk for at least three servings per day was associated with a higher prostate cancer-specific mortality in patients with localized disease (T1/T2 or M0) [67].

### Plant-based Proteins

Soy and soy-based products are rich in protein and isoflavones [39]. Due to their high estrogen activity, isoflavones are characterized as phytoestrogens and exhibit estrogenic effects in prostate tissue [3, 68]. Isoflavones accumulate in the prostate gland, where they exhibit a cytotoxic effect and block the cell cycle, induce apoptosis, inhibit angiogenesis and inhibit 5-alpha-reductase [34, 38, 69]. Genistein is the most abundant isoflavone component in soy and has been shown to inhibit both androgen-dependent and androgen-independent prostate cancer cell growth [34, 69, 70].

The high consumption of soy products is believed to be the reason for the lower incidence of prostate cancer in the Asian population [38].

In a meta-analysis of six case–control and two cohort studies, the consumption of dietary isoflavones showed a significant relationship between increased soybean consumption and reduced prostate cancer risk by 26% [71]. In another meta-analysis of five cohort and eight case–control studies the consumption of non-fermented soybean products was significantly associated with a reduced risk of prostate cancer by 25% [72]. A more recent meta-analysis of fifteen case–control studies, eight cohort studies and seven nested case–control studies reported a lower risk of prostate cancer associated with the intake of daidzein and genistein [73].

#### Carbohydrates

Carbohydrates are the primary fuel for energy production. Depending on their size, carbohydrates are grouped into three classes, including monosaccharides, disaccharides and polysaccharides [3].

Monosaccharides and disaccharides, which are abundant in fruits, dairy products and sugar, are rapidly metabolized in the body. Overconsumption can lead to obesity and hyperinsulinemia, resulting in overproduction of IGF-1 and consequent inflammation, increasing the risk of prostate cancer development and growth [3, 38]. Since insulin is believed to be a growth factor for prostate cancer, it has been hypothesized that reducing carbohydrate intake would result in lower insulin levels, slowing the growth of cancer cells [39]. It has been reported that reducing energy supply in diet modulates numerous processes in cells resulting in an inhibition of carcinogenesis through enhanced apoptosis and programmed cell death processes [74].

In an animal study, xenografted mice implanted with prostate cancer cells, were fed with a no-carbohydrate ketogenic diet. Compared to mice fed with Western diet they had a 15% reduction in weight, a 33% reduction in tumor size and an increase in overall survival. Moreover, it was noticed that mice fed with a no-carbohydrate ketogenic diet had lower IGF-1 levels [75].

Due to practical challenges for humans to refrain from carbohydrates entirely, another animal study examined the impact of low-carbohydrate diet (10–20% kcal carbohydrates) on prostate cancer growth. The results showed that mice consuming low-carbohydrate diets had similar tumor growth and overall survival rates compared to mice consuming no-carbohydrate diets [76].

In human studies, results of investigations examining the relationship between consumption of carbohydrates and high glycemic foods and the risk of prostate cancer have been inconsistent [77–81].

In a prospective study of 3184 adults from the Framingham Offspring cohort, both the quantity and the quality of carbohydrates and sugars were investigated in relation to obesity-related cancers, including prostate, breast and colon cancer [79]. It was noted that higher intake of fruit juice was associated with prostate cancer risk, whereas no association between prostate cancer risk and total consumption of carbohydrate-heavy or sugary beverages was noticed [79].

A study comprising 22,720 men reported that sugar intake from fruit juice was unrelated to the risk of prostate cancer, whereas the intake of sugar-sweetened beverages was related to a high risk [80].

A case–control study of 982 men found that an increased intake of refined carbohydrates, including bagels, rolls, French fries, chocolates, cookies and cakes, was not only associated with an increased risk of prostate cancer, but moreover with a higher risk of aggressive disease [81].

Those inconsistencies may be related to the wide variability in diets in different geographic locations, race, genetic components and food preparation styles [82]. Moreover, those inconsistencies suggest that the quality of carbohydrates, such as refined carbohydrates, might be more relevant for disease development rather than the quantity.

#### Vitamins & Minerals

Vitamins are carbon-containing compounds that are essential to the body for normal growth and function. With the exception of vitamin D, the body cannot produce vitamins. Therefore, humans are required to obtain vitamins from the diet [3]. There are two types of vitamins; the fat-soluble and the water-soluble vitamins. Fat-soluble vitamins include vitamins A, D, E and K, while B-vitamins and vitamin C are water-soluble. Among those, vitamins A, D and E have been investigated regarding their influence on prostate cancer prevention [3, 83].

### Vitamin A

The two forms of vitamin A include pro-vitamin A and pre-formed vitamin A. The pre-formed vitamin A is the active form of vitamin A and is only found in animal food, such as liver [3, 83, 84]. Pro-vitamin A needs to be converted into its active form, which is retinol [83, 84].

In addition to its important role of maintaining healthy vision, vitamin A exhibits antineoplastic properties, such as inhibition of cellular proliferation, induction of apoptosis and anti-oxidant effects [3, 83, 85].

While animal studies indicate that vitamin A and retinoid acids are promising dietary compounds for reducing the risk of prostate cancer, related human studies show inconsistent and conflicting results. The Alpha-Tocopherol, Beta-Carotene Cancer (ATBC) Prevention Study found 23% higher prostate cancer incidence in men receiving 20mg beta-carotene supplements daily [86], while another trial reported no prostate cancer effects at 30mg of beta-carotene [87]. A systematic review and dose-dependent meta-analysis showed that dietary intake of beta-carotenes was not associated with reduced prostate cancer risk [88]. Moreover, beta-carotene supplementation, specifically in smokers, and higher circulating retinol concentrations appeared to be associated with increased risk of prostate cancer [83].

### Vitamin D

Vitamin D is synthesized in the skin in response to sunlight. The primary active form of vitamin D, 1,25 dihydroxyvitamin D3, regulates bone mineralization [3, 83, 84].

However, vitamin D has also been shown to have anti-carcinogenic properties, including promotion of apoptosis, immunomodulation and inhibition of angiogenesis and proliferation [84]. Despite the expected benefit of vitamin D for preventing prostate cancer, the results in literature have been inconsistent and debatable. While some studies indicated that higher vitamin D levels negatively affect prostate cancer status [89, 90], others suggested the exact opposite [83, 91, 92]. Currently, the role of vitamin D in prostate cancer risk remains unclear.

### Vitamin E

Vitamin E is a generic term for two families of fat-soluble compounds with vitamin E activity; tocopherols and tocotrienols [3]. Vitamin E exerts strong antioxidant, anti-inflammatory and anti-cancer effects [3]. It has been suggested that vitamin E slows tumor growth by inhibiting DNA synthesis and induction of apoptotic pathways [39].

A large randomized, placebo-controlled trial examined the effect of supplementation of vitamin E and selenium. It was noted that the combined supplementation of selenium and vitamin E had no effect on reducing the risk of prostate cancer. Surprisingly, vitamin E supplementation alone was associated with an increased risk for prostate cancer [93]. Another study demonstrated that higher.

plasma concentrations of tocopherols in men with prostate cancer recurrence were negatively correlated with serum PSA levels [94].

On the other hand, two observational studies, including the Cancer Prevention Study II Nutrition Cohort and the NIH-AARP Diet and Health Study showed no association between vitamin E supplementation and prostate cancer risk [95, 96].

At this stage, there is no evidence that the supplementation of a single vitamin may offer protection against prostate cancer. However, it is important to mention that vitamins do not occur in isolation in nature, and therefore, supplements may not offer the same therapeutic advantage as consuming a spectrum of nutrients via food.

### Calcium

While current guidelines recommend a daily intake of 1000—1200mg of calcium in men over the age of 50 years, an increased calcium intake of > 1500mg has been associated with an increased risk of prostate cancer [66, 69, 97]. Calcium ions are known to be a key regulator of cell growth, division and differentiation and elevated calcium concentrations are known to promote cell proliferation, especially in the context of prostate cancer [38, 69, 97].

Since the main nutritional source of calcium is a diet rich in dairy products, there is a positive relationship between a high intake of milk, cheese and other dairy products and prostate cancer [5, 38, 97].

Moreover, the intake of dairy products is associated with increased IGF-1 levels, promoting proliferation of prostate cancer cells, and an increased secretion of pro-inflammatory cytokines, such as IL-6, IL-1β and tumor necrosis factor (TNF) alpha [66].

Non-dairy calcium and supplemental calcium are not associated with an increased risk of prostate cancer [2, 66].

#### Phytonutrients

Phytonutrients are chemical compounds generated from secondary plant metabolism, which are beneficial to human health. They are found in various types of food, including fruit, vegetables, grains, herbs or spices and contribute to color, flavor and aroma [98, 99].

High intake of phytonutrients has been associated with lower risk of chronic diseases and mortality, which is attributed to anti-oxidant, anti-inflammatory and anti-cancer effects [98]. Phytonutrients are classified according to different chemical compounds and their properties and are grouped as polyphenols, terpenes, phytosterols, alkaloids and organosulphur compounds [98, 99].

The association of prostate cancer and several phytonutrients is well documented and will be discussed here.

### Lycopenes

Lycopene, a non- vitamin A carotenoid, is a fat-soluble red pigment produced by plants. It is highly abundant in tomatoes, watermelon, papaya or pink grapefruit [38, 98–100]. This pigment has strong anti-oxidant properties and can neutralize reactive oxygen species and organic free radicals produced during the peroxidation process [38].

Further protective effects of lycopene on the prostate gland include the ability to regulate the cell cycle, repair DNA damage and influence the biologic activity of IGF-1 [38].

A systematic review and meta-analysis of 42 publications, of which 25 investigated the impact of dietary lycopene and 18 focusing on the circulating lycopene in relation to prostate cancer risk, reported that both dietary lycopene and circulating concentrations were significantly related to a lower risk of prostate cancer. Moreover, the authors stated that the additional consumption of lycopene by 2mg per day was associated with a lower risk of prostate cancer by 1% [101]. A cohort study showed that healthy men consuming more lycopene were reported to have a 28% lower risk of developing lethal prostate cancer compared to those consuming less [102].

In a randomized controlled trial, 26 men with newly diagnosed and clinically localized prostate cancer were randomly assigned to receive 15mg lycopene twice daily or no supplementation for three weeks before radical prostatectomy. Plasma PSA levels decreased in the intervention group by 18%, while they increased by 14% in the control group [103].

### Catechins

Catechins are flavonoids with strong antioxidant properties and protect cells from damage caused by free radicals. Food sources rich in catechins are green tea, black grapes, apples, pears and berries [99]. Catechins are reported to inhibit prostate cancer growth, induce intrinsic and extrinsic apoptotic pathways, decrease inflammation by inhibiting COX-2 levels and IGF-1-related signalling, and exert antioxidant properties [3, 39].

Special interest in the potential preventative effect of green tea on prostate cancer stemmed from epidemiological observations of the low incidence of prostate cancer in the Asian population [69]. Several studies have demonstrated a beneficial impact of green tea on prostate cancer [104–106]. A large-scale prospective study of 49,920 Japanese men examining the relationship between consumption of green tea and prostate cancer risk, showed that men who drank five or more cups of green tea per day had a lower risk of advanced prostate cancer compared to those, who drank less than one cup per day [106].

### Glucosinolates

Glucosinolates are abundant in cruciferous vegetables, such as in broccoli, cauliflower, kale, Brussels sprouts, cabbage or kale [99].

Indole-3 carbinole is a breakdown product of glucosinolates and has been implicated in a variety of anti-cancer mechanisms, such activating liver detoxification, protecting cells from DNA damage, inducing apoptosis and inhibiting proliferation of prostate cancer cells [2, 19]. Greater consumption of cruciferous vegetables has been shown to be associated with a lower risk of developing aggressive prostate cancer [107, 108]. A prospective study of men with localized prostate cancer showed that those consuming half a cup of cruciferous vegetables per day had a lower risk of recurrence compared to those, who did not consume any [109].

### Tannins

Tannins are a subgroup of polyphenols and are abundant in pomegranate, raspberries and walnuts [2, 99]. Pomegranate in particular has been shown to inhibit the proliferation of prostate cancer cell lines in vitro and to induce apoptosis in a dose-dependent fashion [2, 100]. In human trials, the results have not been convincing.

In a phase 2, randomized double blind study, 68 men were randomized to pomegranate extract vs. placebo for four weeks prior to radical prostatectomy. There was no significant change in the PSA levels compared to placebo [110]. Other placebo-controlled randomized-controlled trials on pomegranate showed no benefit in prostate cancer progression and PSA levels [111, 112].

### Organosulphur Compounds

Allium vegetables, including garlic, onions, leek and chives are rich in organosulphur compounds and flavonols [113–116].

Both in vitro and in vivo studies have shown extensive anti-cancer properties of allium vegetables, including anti-proliferative, anti-inflammatory, anti-oxidant and cytotoxic properties via cell-cycle arrest and induction of apoptosis [113, 116]. A meta-analysis of case–control studies showed a significant reduction in cancer risk with allium vegetables [113, 115]. Consumption of garlic and onion at least once a week is associated with a significant decrease in risk for prostate cancer [116]. One study from China investigating the association between allium vegetables and prostate cancer risk, showed that the consumption of allium vegetables more than 10g/day was associated with a reduced risk of prostate cancer compared to those who consumed less than 2g/day [117].

### Polyphenols

Turmeric derives from the *Curcuma Longa* plant and contains the polyphenol curcumin [118]. Both in-vitro and in-vivo studies have demonstrated the anti-cancer effects of curcumin. The anti-cancer effects of curcumin are based on the inhibition of cancer cell growth and reduced survival, anti-proliferative properties, induction of apoptosis and decreased testosterone production by reduced expression of steroidogenic proteins [118, 119].

Animal studies demonstrated the anti-prostate cancer effects of curcumin. The administration of 500mg/kg of curcumin per day for one month resulted in a 27% delay in tumor growth [120]. In another animal study, the injection of 25-100mg/kg of curcumin resulted in a significant reduction of tumor volume, growth and weight [121].

Since the oral administration of curcumin undergoes rapid metabolism resulting in a narrow systemic distribution, larger doses are required to maintain its therapeutic effects [119]. However, the implementation of nanoemulsions and liposomes demonstrated increased therapeutic efficacy compared to curcumin administered alone, including increased cellular uptake and cytotoxic effects [119, 122].

### Plant-based Diets

While animal-based foods are associated with increased risk of cardiovascular diseases and cancer, plant-based diets reduce the risk of many health conditions and increase survival [123]. Potential mechanisms, through which plant-based diets may reduce the risk of prostate cancer, include both reduced exposure to heterocyclic amines formed during cooking and hormones found in animal food, and through an increased exposure to anti-cancer compounds found in plant food [124]. Plant-based diets are rich in anti-oxidants and anti-inflammatory properties [125]. Since chronic inflammation is associated with oxidative stress and free radical formation enhancing the development of prostate carcinogenesis, plant-based diets may reduce the risk of prostate cancer [124–126].

Adherence to the Mediterranean Diet, which is rich in fruits, vegetables, olive oil, fish and omega-3 fatty acids, was shown to be associated with a great reduction in prostate cancer risk [127].

Asian countries with high consumption of omega-3 fatty acids, soy and green tea-based phytochemicals have lower incidences of prostate cancer compared to countries with high consumption of the ‘Western Diet’ [127].

A systematic review of 32 publications on plant-based diets and incidence and outcomes on prostate cancer showed a lower risk for prostate cancer for men on plant-based diets [123].

Diet recommendations should not be targeted at increasing or decreasing consumption of specific nutrients. Instead a balanced diet with a daily intake of at least 240g of a variety of vegetables is recommended for general health promotion [113].

## Conclusion

The impact of diet and nutrition on prostate cancer and its progression has received tremendous attention over the last decades. A better understanding of the pathogenesis of prostate cancer has demonstrated that inflammation, oxidative stress and the impact of sex hormones play an important role in the initiation and progression of disease. While the impact of several nutrients has shown benefits in animal and in vitro studies, results in humans have been inconsistent, which in part is due to the heterogeneity of studies, diverse research population, geographic location and food quality (organic vs. non-organic), preparation (home made vs. take away) and processing.

Moreover, literature frequently assessed the impact of single nutrients on disease outcome, resulting in disappointing outcomes.

It is important to keep in mind that nutrients, such as vitamins or minerals do not occur in isolation in nature, and therefore, consuming a spectrum of nutrients via food and enjoying a ‘colorful plate’ might render more benefits rather than supplementing single nutrients. Promoting a dietary pattern low in processed meat, dairy products, refined carbohydrates and saturated fats, but high in fruits and vegetables may have beneficial effects on prostate metabolism and inhibit various stages of carcinogenesis through multiple mechanisms.

Adopting a plant-based, antioxidant-rich and an anti-inflammatory diet should be reinforced with patients, who inquire about ways to lessen their risk of prostate cancer.

## Key References

- Aronson WJ, Barnard RJ, Freedland SJ et al.: Growth inhibitory effect of low fat diet on prostate cancer cells: Results of a prospective, randomized dietary intervention trial in men with prostate cancer. *The Journal of Urology* (2010); 183: 345 – 350
  o This study highlights that diets rich in omega-6 fatty acids and trans fatty acids result in increased androgen levels, growth factors, oxidative stress and free radical damage and that a diet rich in omega-3 fatty acids results in significantly slower tumour growth.
- Liss MA, Al-Bayati O, Gelford J et al.: Higher baseline dietary fat and fatty acid intake is associated with increased risk of incident prostate cancer in the SABOR study. *Prostate Cancer and Prostatic diseases* (2019); 22: 244 – 251
  o This is a large, population-based cohort study emphasizing the importance of saturated and trans fatty acids on the risk of development and progression of prostate cancer.
- Aune D, Navaro Rosenblatt DA, Chan D et al.: Dairy products, calcium, and prostate cancer risk: a systematic review and meta-analysis of cohort studies. *American Journal of Clinical Nutrition* (2015); 101: 87 – 117
  o This is a meta-analysis of observational studies demonstrating an increased risk of prostate cancer with high intake of total dairy products, milk, cheese, dietary calcium, low-fat milk and skim milk combined.
- Freedland SJ, Mavropoulos J, Wang A et al.: Carbohydrate restriction, prostate cancer growth, and the insulin-like growth factor axis. *The Prostate* (2008); 68: 11 – 19
  o This study showed that a low-carbohydrate diet in mice resulted in a 33% reduction in tumor size and an increase in overall survival.
- Rowles JL, Ranard KM, Smith JW et al*.*: Increased dietary intake and circulating lycopene are associated with reduced prostate cancer risk: A systematic review and meta-analysis. *Prostate Cancer and Prostatic diseases* (2017); 20: 361 – 377
  o A systematic review and meta-analysis of 42 publications reporting that both dietary lycopene and circulating concentrations were significantly related to a lower risk of prostate cancer.

## Data Availability

No datasets were generated or analysed during the current study.

## Appendix

Table 1

**Table 1:** Summary table of allowed and prohibited food sources

Foods increasing the risk of prostate cancer	Foods reducing the risk of prostate cancer
• Diets rich in Omega-6 fatty acids [13–16] • Red meat [29] • Eggs [44] • Poultry [44] • Dairy products [57–59] • Refined carbohydrates [73]	• Diets rich in Omega-3-Fatty acids [3, 20–25] • Fish rich in Omega-3-Fatty acids [38, 40] • Plant-based proteins [61–65] • Tomatoes [90–92] • Green tea [98] • Cruciferous vegetables [99–101] • Curcumin [110–113] • Allium vegetables [105, 108, 109]

## Abbreviations

*ADT*: Anti-androgen therapy
*ALA*: Alpha-linolenic Acid
*BPA*: Bisphenol A
*COX*: Cyclo-oxygenase
*EPA*: Eicosapentaenoic acid
*IGF*: Insulin-like growth factor
*IL*: Interleukin
*LA*: Linoleic Acid
*LOX*: Lipoxygenase
*PSA*: Prostate-specific antigen
*PUFA*: Polyunsaturated Fatty Acids
*TNF*: Tumor necrosis factor
*VEGF*: Vascular endothelial growth factor
